# Enhancing Accuracy in Breast Density Assessment Using Deep Learning: A Multicentric, Multi-Reader Study

**DOI:** 10.3390/diagnostics14111117

**Published:** 2024-05-28

**Authors:** Marek Biroš, Daniel Kvak, Jakub Dandár, Robert Hrubý, Eva Janů, Anora Atakhanova, Mugahed A. Al-antari

**Affiliations:** 1 Carebot, Ltd., 128 00 Prague, Czech Republic; marek.biros@carebot.com (M.B.); jakub.dandar@carebot.com (J.D.); robert.hruby@carebot.com (R.H.); anora.atakhanova@carebot.com (A.A.); 2 Department of Simulation Medicine, Faculty of Medicine, Masaryk University, 625 00 Brno, Czech Republic; 3 Department of Radiology, Masaryk Memorial Cancer Institute, 602 00 Brno, Czech Republic; 4 Department of Artificial Intelligence and Data Science, Daeyang AI Center, Sejong University, Seoul 05006, Republic of Korea; en.mualshz@sejong.ac.kr

**Keywords:** BI-RADS, breast density, computer-aided diagnosis, deep learning, full-field digital mammography, medical image processing

## Abstract

The evaluation of mammographic breast density, a critical indicator of breast cancer risk, is traditionally performed by radiologists via visual inspection of mammography images, utilizing the Breast Imaging-Reporting and Data System (BI-RADS) breast density categories. However, this method is subject to substantial interobserver variability, leading to inconsistencies and potential inaccuracies in density assessment and subsequent risk estimations. To address this, we present a deep learning-based automatic detection algorithm (DLAD) designed for the automated evaluation of breast density. Our multicentric, multi-reader study leverages a diverse dataset of 122 full-field digital mammography studies (488 images in CC and MLO projections) sourced from three institutions. We invited two experienced radiologists to conduct a retrospective analysis, establishing a ground truth for 72 mammography studies (BI-RADS class A: 18, BI-RADS class B: 43, BI-RADS class C: 7, BI-RADS class D: 4). The efficacy of the DLAD was then compared to the performance of five independent radiologists with varying levels of experience. The DLAD showed robust performance, achieving an accuracy of 0.819 (95% CI: 0.736–0.903), along with an F1 score of 0.798 (0.594–0.905), precision of 0.806 (0.596–0.896), recall of 0.830 (0.650–0.946), and a Cohen’s Kappa (κ) of 0.708 (0.562–0.841). The algorithm achieved robust performance that matches and in four cases exceeds that of individual radiologists. The statistical analysis did not reveal a significant difference in accuracy between DLAD and the radiologists, underscoring the model’s competitive diagnostic alignment with professional radiologist assessments. These results demonstrate that the deep learning-based automatic detection algorithm can enhance the accuracy and consistency of breast density assessments, offering a reliable tool for improving breast cancer screening outcomes.

## 1. Introduction

**Figure 1:**
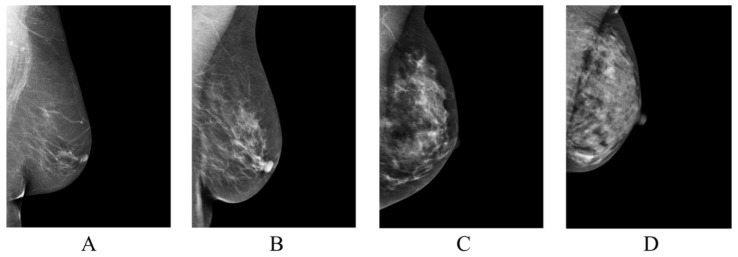
Classification of breast tissue density according to BI-RADS Atlas Fifth Edition classification [4]. Examples of the breast in left mediolateral (LMLO) projection: Class (**A**) = the breasts are almost entirely fatty. Class (**B**) = there are scattered areas of fibroglandular density. Class (**C**) = the breasts are heterogeneously dense, which may obscure small masses. Class (**D**) = the breasts are extremely dense, which lowers the sensitivity of digital mammography.

Population-wide breast cancer screening initiatives have been instrumental in reducing mortality, with high adherence to regular screenings significantly impacting outcomes [1]. Despite significant advancements, breast cancer remains the leading cause of cancer-related deaths among women globally. The effectiveness of current mammography screening protocols has been questioned due to the prevalence of recalls and false positives, which often result in unnecessary biopsy procedures [2]. Breast tissue density is a key determinant in the detection of breast cancer, as it can obscure tumor visibility and is independently linked to a heightened risk of developing breast cancer [3]. To standardize breast density evaluations, the American College of Radiology (ACR) introduced the Breast Imaging-Reporting and Data System (BI-RADS) [4] for breast density assessment (Figure 1). However, the classification system has faced challenges due to the significant interobserver variability among radiologists, leading to inconsistencies and uncertainties in assessments [5,6,7].

Recent advancements in artificial intelligence (AI) and deep learning (DL) have demonstrated the potential to improve diagnostic accuracy in medical imaging [8,9,10]. This study investigates the efficacy of a deep learning-enhanced computer-aided diagnosis (CAD) system in evaluating breast tissue density according to the BI-RADS density classification. The primary objective is to enhance the consistency of breast tissue density evaluation, thereby facilitating improved risk stratification and patient management.

## 2. Background

The emergence of deep learning in healthcare has marked a transformative period in medical imaging, introducing an innovative paradigm for the analysis and interpretation of radiological images [11]. As efforts to achieve uniform and consistent evaluations of breast density intensify, several studies have highlighted the limitations of the current BI-RADS classification. These findings underscore the need for enhanced precision in the guidelines and improved training to ensure dependable density categorization worldwide [12].

One of the first applications of deep learning in breast density assessment was pioneered by Kallenberg et al. [13]. Their study leveraged unsupervised convolutional sparse autoencoders (CSAE) for breast density segmentation and mammographic risk scoring, demonstrating a significant potential for these networks in automating the classification of breast tissue density. Mohamed et al. [14] developed a deep learning system using convolutional neural networks (CNNs), designed to classify mammographic breast density as ‘scattered density’ or ‘heterogeneously dense’. The presented system, trained on 22,000 digital mammogram images from 1427 women, showed promising results, achieving an AUC of 0.9421 initially, which increased to 0.9882 after dataset refinement. Becker et al. [15] expanded the scope of deep learning applications in mammography by evaluating the diagnostic accuracy of an AI-based system not only for BI-RADS density classification but also for quantifying absolute dense tissue percentage. Employing an AI model trained on a dual-center dataset of 3228 mammograms, their approach achieved an AUC of 0.82, demonstrating a high correlation with expert radiologists’ assessments, which ranged from AUCs of 0.77 to 0.87. Similarly, Li et al. [16] applied dilated and attention-guided residual learning techniques for multi-view mammographic density classification. Their method, evaluated on both a clinical dataset and the INBreast dataset, achieved an accuracy of 88.7% and 70.0%, respectively. Furthermore, Deng et al. [17] introduced a novel SE-Attention neural network, integrated using the CNN framework, which was trained on a substantial dataset of 18,157 images from 4982 patients. This approach outperformed traditional models, achieving accuracy as high as 92.17% on the Inception-V4-SE architecture. Lastly, Wu et al. [18] explored the application of multi-column deep CNNs in classifying breast density using 201,179 screening mammograms. Their model achieved a top-1 accuracy of 76.7%, a top-2 accuracy of 98.2%, and a macAUC of 0.916, demonstrating the efficacy in handling large-scale, clinically realistic datasets.

Detailed information on the comparable studies, including dataset specifics, methodologies, and performance metrics, are provided in Table A1.

## 3. Materials and Methods

### 3.1. Software

**Figure 2:**
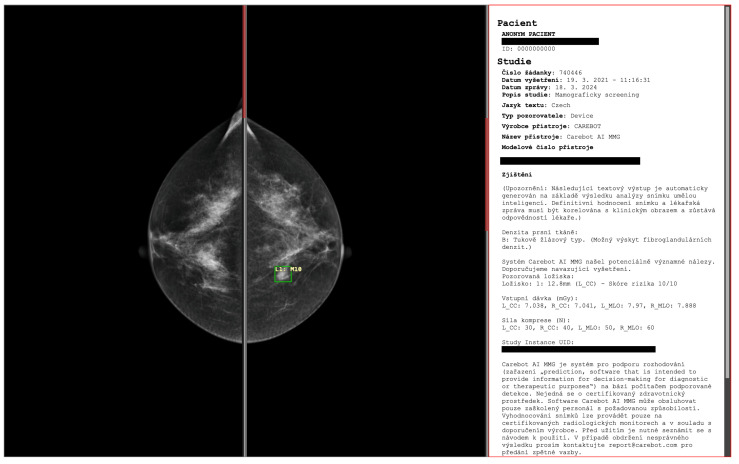
The proposed deep learning-based automatic detection algorithm (DLAD, Carebot AI MMG v2.2; Prague, Czech Republic), implemented in CloudPACS v2.12.28 (OR-CZ spol. s r.o.; Moravská Třebová, Czech Republic). Digital mammography of a 47-year-old woman with lower-density breasts (BI-RADS B).

The proposed deep learning-based automatic detection algorithm (DLAD, Carebot AI MMG v2.2; Prague, Czech Republic) analyzes full-field digital mammography (FFDM) studies in the standard left (LCC) and right craniocaudal (RCC), and left (LMLO) and right mediolateral oblique (RMLO) view. The images are initially directed to a preprocessing module, which eliminates extraneous components from the images and implements image filtering techniques. The images are then classified by the DLAD’s multi-class classifier, adhering to the BI-RADS breast density classification standards. Designed to augment the decision-making process in screening clinical practice, the DLAD is designed for seamless integration with picture archiving and communication systems (PACS) and DICOM viewers (Figure 2) using DICOMweb and DIMSE protocols.

### 3.2. Train Data

To determine the ground truth for our training data, we have established a team of 10 breast radiologists with 2 to 27 years of experience in mammography interpretation, including 7 board-certified radiologists and 3 junior radiologists without board certification. Each of the 8295 mammography studies (33,180 images) was randomly assigned to two radiologists to evaluate the presence of benign or malignant lesions, and suspect microcalcifications, and to determine breast density according to BI-RADS breast density classification. A consensus between the two radiologists was required to establish the ground truth.

**Figure 3:**
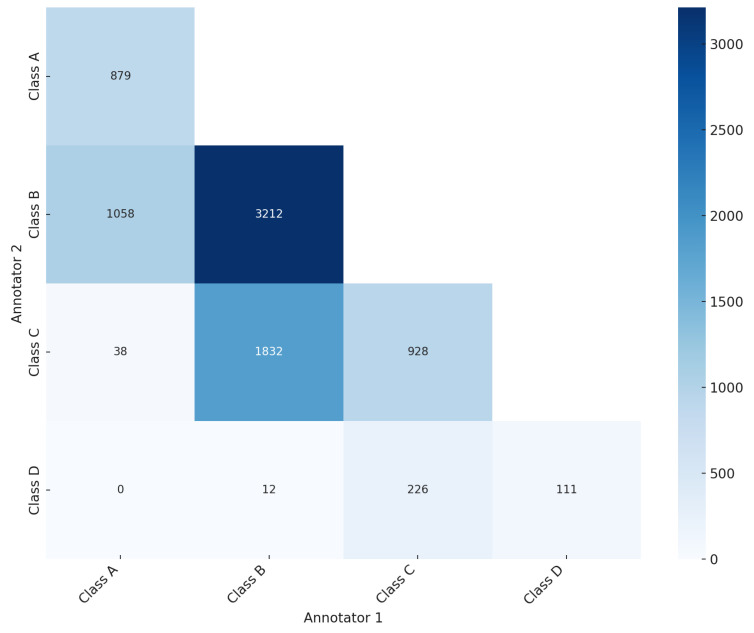
Confusion matrix showing the agreement and disagreement among annotators in breast density assessment according to BI-RADS breast density classification.

As illustrated in the confusion matrix (Figure 3), the ground truth was established for 5130 mammography studies (20,520 images, 61.84%), while for 3165 studies (12,660 images, 38.16%) the ground truth was not reached. Of the 5130 studies with ground truth, 879 mammography studies (3516 images) were classified as BI-RADS class A, 3212 studies (12,848 images) as BI-RADS class B, 928 studies (3712 images) as BI-RADS class C, and 111 studies (444 images) as BI-RADS class D (Table 1). The significant level of disagreement highlights the challenge of consistent mammographic density interpretation, thereby illustrating the potential value of CAD systems in improving assessment accuracy. This aligns with findings from previous studies [19,20,21], which promote the integration of deep learning models to mitigate variability and enhance evaluation accuracy.

### 3.3. Model Architecture

**Figure 4:**
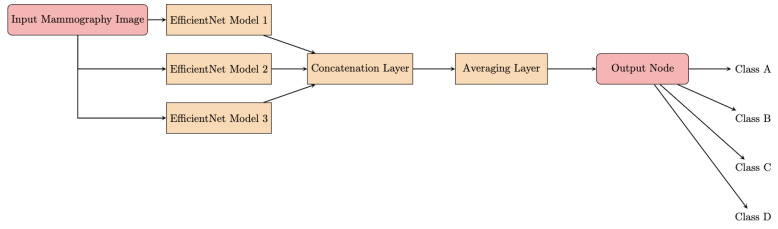
Flowchart of the model soup architecture.

The architecture of the proposed DLAD leverages the model soup approach [22], which involves creating an ensemble model by aggregating weights from multiple independently fine-tuned EfficientNet [23] models. This methodology produces a single model that incorporates the collective attributes of several configurations, each characterized by unique optimizations of the hyperparameters [24]. The foundation of the DLAD architecture is represented by EfficientNet, a scalable convolutional neural network (Figure 4). A major innovation of the network architecture is the methodical scaling of the network’s dimensions—depth, width, and image resolution—to achieve an optimal balance between computational efficiency and model performance. The scalability of EfficientNet enables adaptation to varying dataset characteristics and analytical objectives.

### 3.4. Test Data

This multicentric study involves the retrospective evaluation of full-field digital mammography studies from three independent sites: Institution 1 (EUC Mamocentrum Brno) and Institution 2 (Hospital Šumperk), both specializing in screening mammography, and Institution 3 (Masaryk Memorial Cancer Institute), a comprehensive oncology facility offering both screening and diagnostic mammography, including post-surgical mammograms. A total of 122 mammography studies (488 images) were acquired: 60 mammography studies (240 images) were collected from Institution 1 using GE Senographe Essential, 28 studies (112 images) from Institution 2 using GE Senographe Essential, and 34 studies (136 images) from Institution 3 using Hologic Selenia Dimensions and Siemens Healthineers MAMMOMAT Revelation (Table 2).

All collected images are full-size, with variable dimensions depending on the source institution and mammography machine. All images were exported in the standard Digital Imaging and Communications in Medicine (DICOM) format, modality MG. The images are uncompressed, maintaining the full resolution and quality for accurate analysis. The data were obtained from the referral centers in an anonymized form, preventing any retrospective identification of patients, in compliance with Regulation (EU) 2016/679 of the European Parliament and the Council. Given this, Carebot Ltd. does not have access to any additional clinical information about the patients.

### 3.5. Ground Truth

The ground truth was established by a consensus of two board-certified radiologists with 13 and 27 years of experience, respectively (Table 3). Consensus was reached in 72 mammography studies (288 images), whereas in 50 studies, there was a disagreement on the BI-RADS breast density category. This resulted in the ground truth not being determined for these studies, and these mammography studies were excluded from the study.

Of the 72 mammography studies (288 images) with ground truth, 33 studies (132 images) were obtained from Institution 1, 15 studies (60 images) from Institution 2, and 24 studies (96 images) from Institution 3 (Table 4).

Regarding the BI-RADS density category, 18 mammography studies (72 images) belonged to BI-RADS class A, 43 studies (172 images) to BI-RADS class B, 7 studies (28 images) to BI-RADS class C, and 4 studies (16 images) to BI-RADS class D (Table 5).

### 3.6. Reader Study

The DLAD analyzes mammography studies in standard projections (CC and MLO) and classifies them according to the ACR BI-RADS Atlas Fifth Edition (class A/B/C/D). The performance of the DLAD evaluated against ground truth is then compared with that of five individual radiologists with varying experience (Table 6).

### 3.7. Statistical Analysis

We conduct a rigorous statistical analysis to evaluate the performance of each method—the proposed DLAD and assessed radiologists in multi-reader study—in classifying BI-RADS breast density. The analysis focuses on key metrics, including accuracy, F1 score (macro-averaged), precision (macro-averaged), recall (macro-averaged), and Cohen’s Kappa (κ) to assess the strength of agreement [25]. As all images were evaluated by all assessed radiologists, we use a bootstrapping method, which involves resampling the test data 1000 times using randomly selected subsets and calculating the metrics for each sample to estimate the 95% confidence intervals (*CI*) for the statistical metrics.

To evaluate the statistical significance of the differences in accuracy between the DLAD and the assessed radiologists, we calculate *p*-values using McNemar’s test. The null hypothesis ($H_{0}$)—stating that there is no difference in performance between DLAD and the radiologists—is tested against an alternative hypothesis ($H_{1}$), which suggests that there is a statistically significant difference in performance (*p*-value < 0.05) and that the strength of agreement of each method with the ground truth is at least moderate (κ > 0.41). Rejection of $H_{0}$ in favor of $H_{1}$ would imply both a statistically significant difference and a clinically relevant level of agreement with the ground truth for either method. Alternatively, failing to reject $H_{0}$ (*p*-value ≥ 0.05) indicates no statistically significant difference in performance, and a κ value ≤ 0.41 for each method suggests that the level of agreement with the ground truth is less than moderate.

## 4. Results

**Figure 5:**
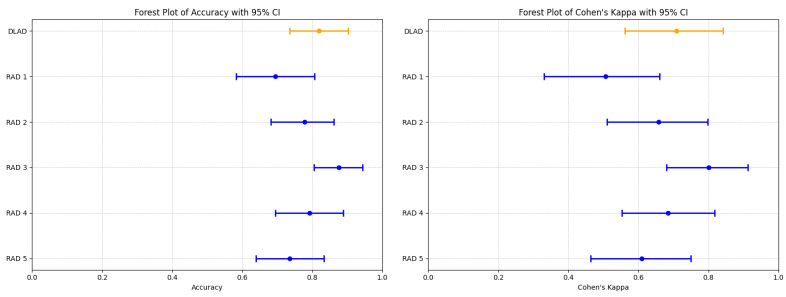
Forest plots illustrating the performance comparison between the proposed DLAD (Carebot AI MMG v2.2) and assessed radiologists (RAD 1–RAD 5) in terms of Accuracy and Cohen’s Kappa (κ). Orange lines indicate DLAD, blue lines indicate RAD 1–RAD 5.

The proposed deep learning-based automatic detection algorithm (DLAD, Carebot AI MMG v2.2) demonstrated notable performance in classifying mammography studies according to BI-RADS categories. Specifically, the DLAD model correctly classified 17 studies as BI-RADS class A, 33 as class B, 6 as class C, and 3 as class D, resulting in an overall accuracy of 0.819 (95% CI: 0.736 to 0.903), and a Cohen’s Kappa (κ) of 0.708 (95% CI: 0.562–0.841), highlighting a substantial agreement with the consensus ground truth (Table 7, Figure 5). The model achieved an F1 score of 0.798 (95% CI: 0.594–0.905), precision of 0.806 (95% CI: 0.596–0.896), and recall of 0.830 (95% CI: 0.650–0.946 (Table 8).

**Figure A1:**
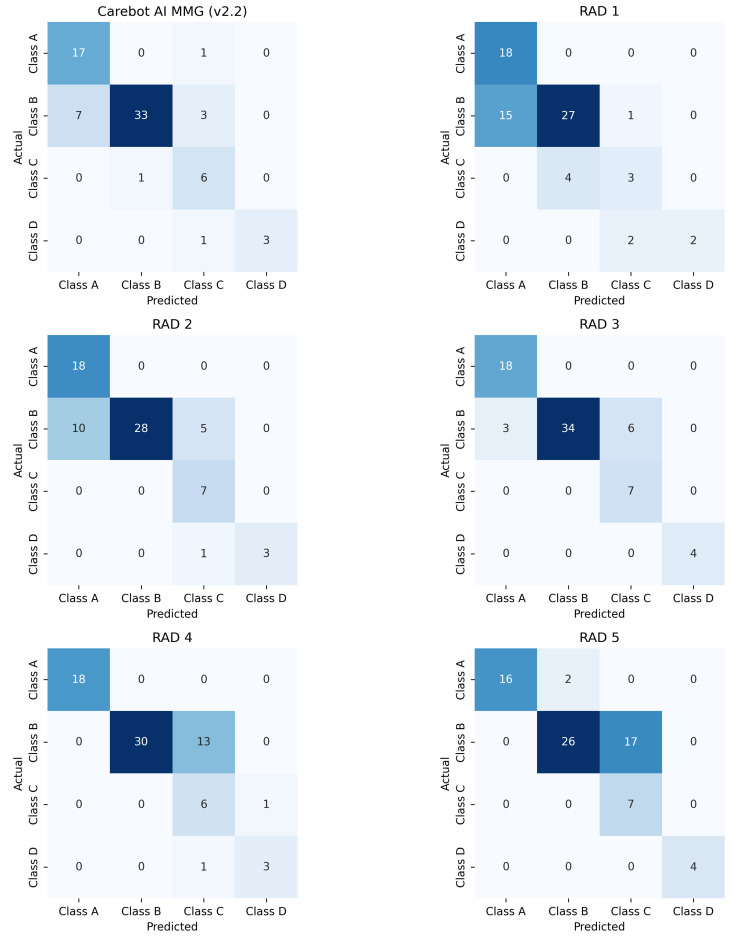
Confusion matrix for the proposed DLAD (Carebot AI MMG v2.2) and the assessed radiologists.

Comparatively, the radiologists’ performance varied (Figure A1), with RAD 3 achieving the highest accuracy of 0.875 (95% CI: 0.805–0.944) and a κ of 0.800 (95% CI: 0.680–0.912), closely aligning with the DLAD model’s performance metrics. The statistical analysis did not reveal a significant difference in accuracy between the DLAD and the radiologists, as indicated by the *p*-values (RAD 1: 0.052, RAD 2: 0.606, RAD 3: 0.423, RAD 4: 0.823, RAD 5: 0.327). Additionally, the κ differences suggest that the level of agreement between the DLAD model and the radiologists is not significantly different, underscoring the model’s competitive diagnostic alignment with professional radiologist assessments.

**Figure 6:**
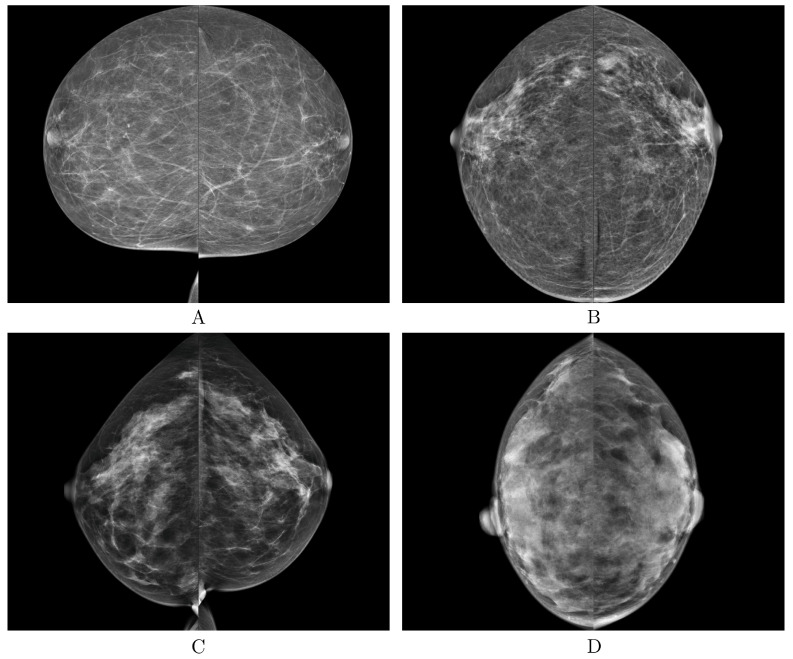
Examples of the correct predictions made by the deep learning-based automatic detection algorithm (DLAD, Carebot AI MMG v2.2) for each breast density BI-RADS class (**A**–**D**).

The proposed DLAD achieved robust agreement with the ground truth, as evidenced by its substantial κ value, and high accuracy in classifying mammography studies according to the BI-RADS scale, also in a multicenter validation involving images representing diverse populations and sourced from three different manufacturers of mammography X-ray machines (GE Senographe Essential, Hologic Selenia Dimensions, and Siemens Healthineers MAMMOMAT Revelation; Figure 6).

**Figure 7:**
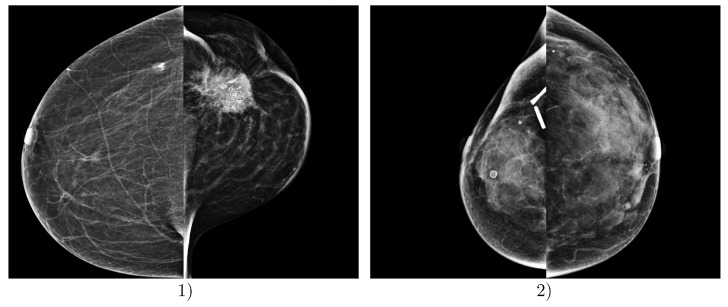
Examples of incorrect predictions made by the deep learning-based automatic detection algorithm (DLAD, Carebot AI MMG v2.2). Example (1) shows a patient FFDM image with ground truth BI-RADS class A, which was incorrectly assessed by the DLAD as BI-RADS class C, due to the prominent malignant lesion in LCC projection. Example (2) shows a patient FFDM image with ground truth BI-RADS class D, which was incorrectly assessed by the DLAD as BI-RADS class C, due to the post-surgical changes and visible metallic density artifacts in the right breast. Both mammography studies are from Institution 3, e.g., the oncology center.

The most problematic images for classification, i.e., those where the proposed DLAD most frequently misclassified mammography studies according to BI-RADS density categories, involved images from Institution 3. In particular, images containing significant malignant lesions, metal artifacts, clips, and other dimensional factors and objects present in the scans were misclassified (Figure 7).

## 5. Discussion

In this study, we explored the potential of leveraging a deep learning-based automatic detection algorithm (DLAD) to enhance the consistency and accuracy in determining breast tissue density according to the BI-RADS classification, an endeavor aiming at facilitating more precise risk estimation and augmenting patient care. The DLAD model, based on the model soup architecture, achieved notable accuracy, demonstrating a significant advancement in automated breast density classification. For individual classes, the DLAD achieved robust levels of accuracy and Cohen’s Kappa across BI-RADS breast density categories, correctly classifying 17 studies as BI-RADS class A, 33 as class B, 6 as class C, and 3 as class D. This resulted in an overall accuracy of 0.819 (95% CI: 0.736–0.903) and a Cohen’s Kappa (κ) of 0.708 (95% CI: 0.562–0.841), highlighting a substantial agreement with the consensus ground truth. The proposed DLAD could serve as substantial support in the evaluation process, introducing an additional layer of analysis that would work in tandem with the expertise of radiologists to analyze mammography images. The notable interobserver variability in mammographic density assessments, as evidenced in Section 3.2, highlights the challenge of achieving consistent evaluations and underscores the need for more objective and automated assessment methods, including the determination of ground truth.

The implications of our findings extend to risk-based screening, where accurate density assessments are crucial for determining appropriate follow-up methods. The statistical analysis did not reveal a significant difference in accuracy between DLAD and the radiologists, as indicated by the *p*-values (RAD 1: 0.052, RAD 2: 0.606, RAD 3: 0.423, RAD 4: 0.823, RAD 5: 0.327), suggesting that the DLAD’s performance is competitively aligned with that of human experts. Furthermore, the κ differences and the associated κ strength of agreement underline the DLAD model’s comparable diagnostic agreement with the professional radiological assessments. Overall, the robustness of our findings is supported by the diversity of image sources, i.e., multiple mammography X-ray machines, and patient selection in our study, which included mammography studies from a variety of institutions with different focuses, including screening and diagnostic centers. This diversity ensures that the performance of the DLAD model is validated under a wide range of real-world conditions, confirming its applicability and effectiveness in a variety of clinical settings.

### Limitations

Despite the promising results of the proposed DLAD in the evaluation of breast tissue density evaluation, our study faces several limitations that must be acknowledged. Firstly, the distribution of breast densities in our dataset did not reflect the prevalence in the general population [26,27]. This discrepancy could impact the generalizability of our results and indicate a need for more representative and extensive sample selection in future studies. Moreover, while our research aligns with the evolving role of AI in breast cancer risk prediction, the limited number of mammograms evaluated—particularly for the BI-RADS class D—presents significant limitations. These factors restrict the robustness of our conclusions and underscore the necessity for future research to encompass larger, more diverse datasets. This expansion would enable a more comprehensive evaluation of DLAD’s effectiveness across the spectrum of breast densities encountered in broader population samples. Additionally, exploring technical solutions for more objective image analysis remains a critical area for further research, aiming to minimize subjectivity in breast density assessments and enhance the predictive accuracy of risk models. Nonetheless, DLAD’s main challenges were inaccuracies in classifying mammography studies with post-surgical changes or artifacts.

## 6. Conclusions

This study demonstrated the deep learning-based automatic detection algorithm’s (DLAD) potential to improve the consistency and accuracy of breast tissue density classification per BI-RADS categories, aiming to refine risk stratification and patient care. Achieving notable accuracy, the DLAD model could significantly support radiologists by providing an additional analytical layer for mammography image evaluation. Given the prevalent interobserver variability in density assessments, our findings underscore the urgency for more objective, automated methods to ensure consistent evaluations.

## Figures and Tables

**Table 1:** Distribution of the proposed deep learning-based automatic detection algorithm (DLAD, Carebot AI MMG v2.2) training data across BI-RADS breast density categories (A, B, C, D).

BI-RADS Category	$n_{\mathrm{STUDY}}$/$n_{\mathrm{IMAGES}}$
Class A	879/3516
Class B	3212/12,848
Class C	928/3712
Class D	111/444
**Total**	**5130/20,520**

**Table 2:** Distribution of acquired test data by institution and specific mammography X-ray machine type, including the number of mammography studies and images.

Institution	Mammography X-ray Machine	$n_{\mathrm{STUDY}}$/$n_{\mathrm{IMAGES}}$
Institution 1	GE Senographe Essential VERSION ADS 54.20	60/240
Institution 2	GE Senographe Essential VERSION ADS 55.31.10	28/112
Institution 3	Hologic Selenia Dimensions	27/108
	Siemens Healthineers MAMMOMAT Revelation	7/28
**Total**		**122/488**

**Table 3:** Ground truth annotators and their corresponding experience.

ID	Experience
GT 1	Head of the radiology department of screening center, 27 years of experience, board-certified
GT 2	Head of the radiology department of oncology hospital, 13 years of experience, board-certified

**Table 4:** Refined distribution of test data by institution and mammography X-ray machine type after ground truth assessment, including the number of mammography studies and images.

Institution	Mammography X-ray Machine	$n_{\mathrm{STUDY}}$/$n_{\mathrm{IMAGES}}$
Institution 1	GE Senographe Essential VERSION ADS 54.20	33/132
Institution 2	GE Senographe Essential VERSION ADS 55.31.10	15/60
Institution 3	Hologic Selenia Dimensions	20/80
	Siemens Healthineers MAMMOMAT Revelation	4/16
**Total**		**72/288**

**Table 5:** Distribution of test data across BI-RADS breast density categories (A, B, C, D) after ground truth assessment.

BI-RADS Category	$n_{\mathrm{STUDY}}$/$n_{\mathrm{IMAGES}}$
Class A	18/72
Class B	43/172
Class C	7/28
Class D	4/16
**Total**	**72/288**

**Table 6:** List of radiologists (RAD 1–RAD 5) participating in the multi-reader study alongside their respective experience levels.

ID	Experience
RAD 1	2 years of experience, without board certification
RAD 2	2 years of experience, without board certification
RAD 3	4 years of experience, without board certification
RAD 4	7 years of experience, board-certified
RAD 5	8 years of experience, board-certified

**Table 7:** Comparison of Accuracy and Cohen’s Kappa (κ) between DLAD (Carebot AI MMG v2.2) and radiologists (RAD 1–RAD 5).

ID	Accuracy (95% CI)	*p*-Value	κ (95% CI)	κ Agreement [25]	κ Difference
**DLAD**	**0.819 (0.736–0.903)**	**-**	**0.708 (0.562–0.841)**	**Substantial**	**-**
RAD 1	0.694 (0.583–0.806)	0.052	0.506 (0.331–0.661)	Moderate	0.203
RAD 2	0.778 (0.681–0.861)	0.606	0.658 (0.511–0.798)	Substantial	0.053
RAD 3	0.875 (0.805–0.944)	0.423	0.800 (0.680–0.912)	Substantial	−0.094
RAD 4	0.792 (0.694–0.889)	0.823	0.684 (0.553–0.818)	Substantial	0.026
RAD 5	0.736 (0.639–0.833)	0.327	0.610 (0.464–0.749)	Substantial	0.099

**Table 8:** Comparison of F1 Score, Precision, and Recall between DLAD (Carebot AI MMG v2.2) and radiologists (RAD 1–RAD 5).

ID	F1 Score (95% CI)	Precision (95% CI)	Recall (95% CI)
**DLAD**	**0.798 (0.594–0.905)**	**0.806 (0.596–0.896)**	**0.830 (0.650–0.946)**
RAD 1	0.641 (0.412–0.789)	0.729 (0.425–0.854)	0.639 (0.458–0.807)
RAD 2	0.782 (0.600–0.879)	0.795 (0.633–0.873)	0.850 (0.697–0.938)
RAD 3	0.877 (0.782–0.941)	0.849 (0.764–0.923)	0.948 (0.915–0.976)
RAD 4	0.754 (0.549–0.861)	0.762 (0.571–0.865)	0.826 (0.630–0.938)
RAD 5	0.781 (0.700–0.855)	0.805 (0.750–0.858)	0.873 (0.816–0.920)

## Data Availability

Data from this study can be provided by Carebot, Ltd., to independent researchers. Please contact the author for more information, if required.

## Abbreviations

The following abbreviations are used in this manuscript:

DLAD	deep learning-based automatic detection algorithm
CAD	computer-aided diagnosis
CNN	convolutional neural network
BI-RADS	Breast Imaging-Reporting and Data System
CI	confidence interval

## Appendix A

**Table A1:** Summary of key studies on breast density classification using deep learning approaches.

No.	Authors	Key Contribution	Datasets	Methodology	Performance Metrics
[13]	Kallenberg et al.	Unsupervised deep learning for breast density segmentation and mammographic risk scoring.	Three clinical datasets; details not fully specified.	Convolutional Sparse Autoencoders (CSAE); Unsupervised feature learning.	Pearson correlation for PMD: 0.85
[14]	Mohamed et al.	Classifying mammographic breast density categories between “scattered density” and “heterogeneously dense”.	22,000 digital mammogram images from 1427 women.	CNN with transfer learning and training from scratch.	AUC: 0.9421 when trained from scratch; AUC increased to 0.9882 after refining the dataset.
[15]	Becker et al.	Evaluate diagnostic accuracy of a multipurpose image analysis software.	Dual-center retrospective study of 3228 mammograms; external validation with a publicly available dataset.	Artificial neural network trained on retrospective and prospective cohort data.	AUC: 0.82; comparable to experienced radiologists (AUC: 0.77–0.87).
[16]	Li et al.	Multi-view mammographic density classification using dilated and attention-guided residual learning.	Two datasets: a clinical dataset and the INBreast dataset.	Dilated and attention-guided residual learning	Accuracy: 88.7% on the clinical dataset, 70.0% on the INBreast dataset.
[17]	Deng et al.	SE-Attention neural networks for breast density classification.	18,157 images from 4982 patients from Shanxi Medical University.	SE-Attention integrated into a CNN framework.	Enhanced performance over traditional models with accuracies reaching up to 92.17% for Inception-V4-SE.
[18]	Wu et al.	Deep learning for automated breast density classification in over 200,000 screening mammograms.	201,179 exams, containing 19,939 class 0, 85,665 class 1, 83,852 class 2, and 11,723 class 3 exams.	Multi-column deep convolutional neural networks (CNNs).	Top-1 accuracy: 76.7%, Top-2 accuracy: 98.2%, macAUC: 0.916.
